# A cost benefits analysis of the adoption of system of rice intensification: Evidence from smallholder rice farmers within an innovation platform in Oluch irrigation scheme, Kenya

**DOI:** 10.1371/journal.pone.0290759

**Published:** 2024-01-02

**Authors:** Matilda A. Ouma, Luke O. Ouma, Justus M. Ombati, Christopher A. Onyango

**Affiliations:** 1 School of Agricultural and Food Sciences, Jaramogi Oginga Odinga University of Science and Technology, Bondo, Kenya; 2 Department of Agricultural Education and Extension, Egerton University, Egerton, Njoro, Kenya; 3 Biostatistics Research Group, Population Health Sciences institute, Newcastle University, Newcastle upon Tyne, United Kingdom; Shahjalal University of Science and Technology, BANGLADESH

## Abstract

In the wake of climate change and dwindling natural resources, system of rice intensification has been fronted as an approach to improve rice production in several countries. Besides the benefits such as improved rice productivity, reduced water usage that have widely been observed, there is need to quantify the economic benefits of system of rice intensification accrued to farmers, thereby promoting it as an innovation to improve livelihoods of rice farmers. This aim of this paper is to quantify the economic benefits of undertaking SRI among smallholder rice farmers. We introduced SRI among smallholder farmers in a rural setting in western Kenya, Oluch irrigation scheme, through an innovation platform approach. Over the period of four years (2016–2019), we quantify the benefits accrued to the uptake of the technology among adopters of the technology. Our comparisons are in reference to a baseline study conducted prior to the full-scale promotion of SRI in the study area. Our study findings reveal that the uptake of specific SRI practices increased by at least 30–80%, and acreage under rice farming increased by 50%. Besides, SRI required more production costs per acre (63% increase), although SRI had at least 28.6% higher return per shilling invested. Our findings underscore previous results in the literature that SRI is associated with not only productivity but also economic benefits justifying the need for scaling especially among smallholder farmers. Nonetheless, efficient approaches to scaling such promising technologies are necessary to enhance productivity and subsequently improve livelihoods.

## 1. Introduction

Rice (*Oryza sativa L*.) productivity in Africa continues to lag below its potential, despite various concerted efforts by governments and the research community. Today, rice is the second most important source of calories after maize for households in sub-Saharan Africa (SSA); the demand for rice is expected to grow by at least 33% between 2018–2026 [1]. Further, it is projected that within the next decade sub-Saharan Africa will be the largest net importer of rice. In Kenya, rice is the third most important food crop with marked fluctuations in productivity in recent years [2]. Here, a single irrigation scheme, Mwea irrigation scheme, accounts for over 80% of the rice produced locally, which is mainly undertaken under conventional rice farming practices [2–4].

System of rice intensification (SRI) is a technology originally developed in Madagascar in the 1980s that is increasingly popular in many rice producing countries as a means of attaining higher yield under minimal resources. Precisely, it comprises the following practices; early transplanting; single widely spaced plants; early and regular (mechanical) weeding; controlled water management (intermittent irrigation); application of manure; no use of herbicides [5]. These environmentally friendly agronomic practices have been associated with higher yields, efficient use of fertilizers, and a decrease in water use in several countries including China, Madagascar, Kenya, Ghana, Thailand, Tanzania [5–8]. Nevertheless, there is still limited evidence of the scale of its adoption and its practice is still a subject of debate [5].

Despite the observed benefits such as the potential increase in rice yield and reduction in water use, the economic benefits of SRI are especially of interest because technological innovations are earmarked to improve livelihoods. Indeed some studies have previously aimed to quantify the economic benefits of SRI in some areas where it has been introduced in Africa. In Kenya, Ndiiri *et al*. compared SRI and conventional methods of rice cultivation and found a 33% increase in gross revenue from SRI yield over conventional farming yields [2]. Kaloi *et al*. reported a 58.3% increase in gross revenue following SRI [9]. Elsewhere, Denkiyrah has also demonstrated profitability of SRI over conventional approaches in a Ghanian setting [10]. In other regions, some authors have reported improved farmer returns by over 400% under SRI through increased yield and reduced costs, while others report that productivity and economics of rice under SRI does not always outperform other methods [11].

One potential concern is that studies SSA have often been conducted at a single sites within a country, often rely on small subset of farmers and use data for a single season. Many published findings in SSA are based on small trials involving a few farmers (typically less than 100) at the point of introduction of the SRI technology. Further, we acknowledge that there are important regional specific value chain differences that may account for variations in achieved benefits. In Kenya for instance, all studies so far about SRI have been undertaken at a single irrigation scheme and quantitatively quantified economic benefits even from the same study area are varied. Ndiiri *et al* for instance undertook a benefit-cost analysis of paddy rice under the system of rice intensification in Mwea, Kenya and reported a 33% increase in yield on average over two seasons [2]. We also note conflicting findings of some Kenyan findings such as increased costs of production attributed to SRI [9], when some larger studies that report reduced costs [12].

We aimed to quantify the benefits that will be accrued to farmers over a four-year period following the introduction of SRI and the presence of an innovation platform for networking and capacity building to support the uptake of the technology. Especially, we focus on a region (Oluch irrigation scheme in Homabay county) where SRI is introduced using a different strategy [13] to that used to first introduce it in Mwea irrigation scheme where all major studies on SRI in Kenya have taken place [14]. In this paper, we describe the uptake of SRI and changes in production in Oluch irrigation scheme during the period of the study. Thereafter we estimate the cost-benefits from SRI over conventional farming prior to the uptake of SRI.

## 2. Methods

### 2.1 Study area

**Fig 1:**
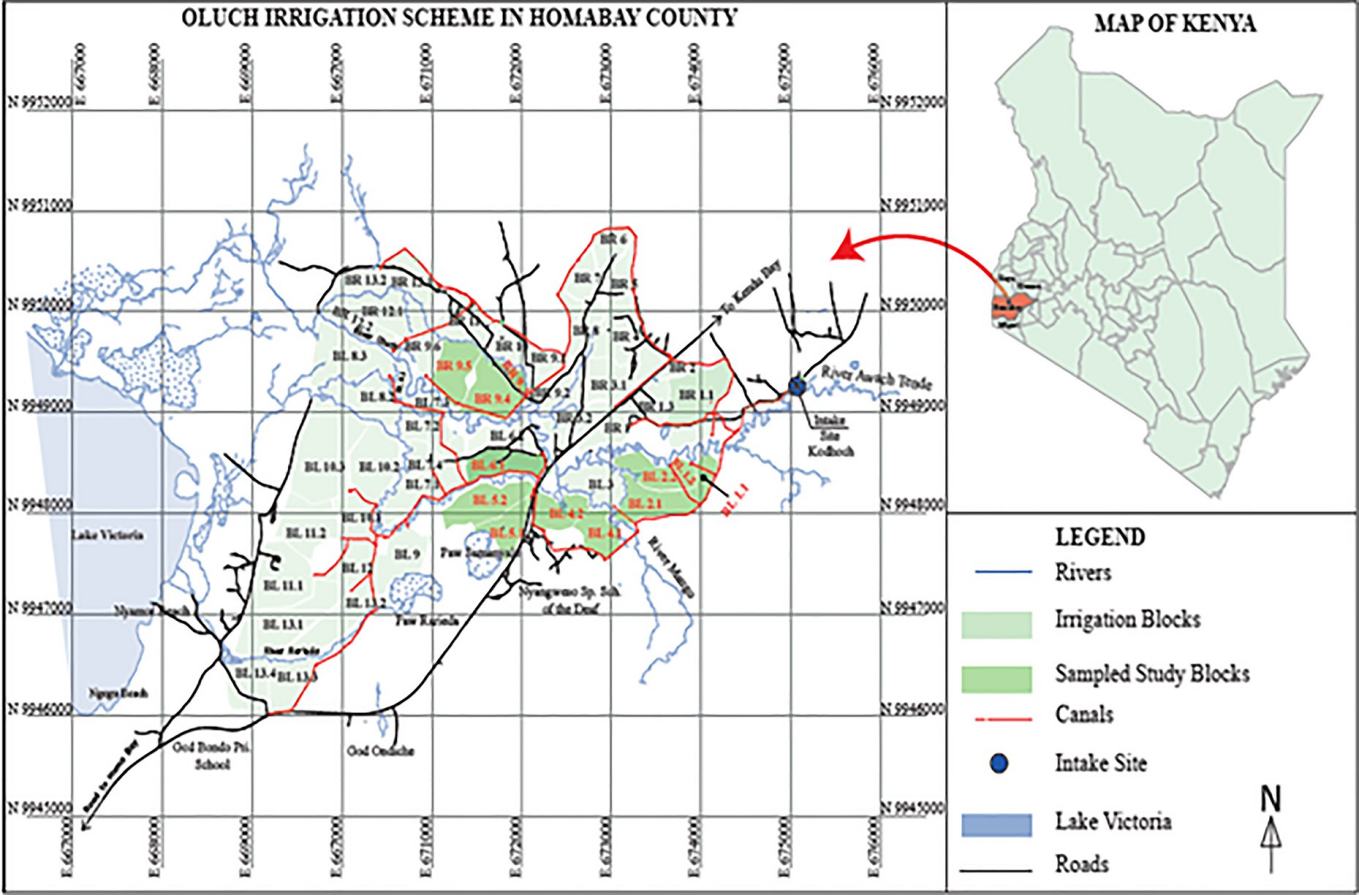
Map showing location of Oluch irrigation scheme.

This study was undertaken at Oluch irrigation scheme, Rangwe sub-county, Homabay county, Kenya (Fig 1) between 2016 and 2019. The scheme was established with funding from African development bank [15] and is organised into 53 irrigation blocks with an acreage of 1308ha. Currently, only half or the total land is under irrigation. Subsistence farming here is very common, but is characterised by very low productivity despite the availability of massive irrigation infrastructure. SRI has previously been introduced here by government extensionists but was hardly adopted prior to the initiation of our study [8].

### 2.2 Study design

The primary study within which the current study is embedded follows an action research design. Precisely, an innovation platform (IP) was designed to spearhead the uptake of system of rice intensification among smallholder farmers. Prior to the formation of this IP, a baseline study involving one hundred farmers was conducted to understand the challenges experienced by farmers, the degree of adoption of SRI practices if any, and coping strategies. Findings of the baseline study have been published by Ouma *et al* (2020) [8]. It is on the basis of the baseline findings by Ouma *et al* that an IP was formed to facilitate action learning process and the use of SRI.

#### Description of the IP

The Innovation Platform was established through an inaugural workshop by involving key stakeholders (farmers, government service providers, traders, KOSFIP, private organizations and technical people) along the rice value chain in Oluch Irrigation Scheme. Stakeholder analysis was conducted to determine the actors with significant influence on the success of SRI technology intervention in smallholder rice production in Oluch Irrigation Scheme. The roles of stakeholders represented in the IP is as shown in Table 1. Rice producers were the primary actors largely represented by smallholder farmers and farmer organizations within Oluch. As producers, farmers formed the first link in the rice value chain whose major roles encompassed rice production, use of production resources and information exchange.

**Table 1:** Stakeholders participating in the innovation platform.

Participant	Attributes	Interest
Smallholder rice farmers	Producers	Learning about SRI and rice production
KALRO, IFDC, Institutions of higher learning	Public research	Adaption of SRI practices to local context
County- extension and KOSFIP	Public extension	Promote SRI practices
LBDA, traders/vendors, private millers, transporters	Public and private output market and intermediaries	Purchase Processor
AfriTech, Baraka, Bayer Co. Agro-dealers, KFA	Public and private input market suppliers	Input supply
NIB/NIA, IWUA, Local Administration	Public policy By-Laws	Promote irrigation for agriculture
AFC, ROSKAS, Muungano Micro-credit society	Private credit institutions	Advance loans and credit to farmers
KOFDEG	Local NGO	Promotion of agriculture
Researchers (MAO)	Academic research	Interactive learning

The IP comprised 24 farmers representative of all the irrigation blocks where rice farming was undertaken at Oluch, and 17 other different stakeholders/important actors in the local rice value chain. A single irrigation block was unanimously selected for establishment of the IP demonstration plot by farmers and other workshop participants. Its suitability was based on the following criteria: ease of accessibility by farmers from other sampled study blocks, its geographical positioning including close proximity to the main highway, Oluch irrigation water user’s association (OL-IWUA) building for hosting IP forums and a nearby Nyangweso market.

Farmers recruited the IP learnt from the various IP stakeholders and took part in establishing and running SRI demonstration plots. Later, these farmers alongside government extensionists trained other farmers within the irrigation scheme on SRI practices. All aspects relating to the SRI implementation from field preparation to mechanized harvesting were illustrated by the lead researcher (MAO).

### 2.3 Data collection & Statistical analysis

Data for this study were obtained using questionnaires, focus group discussions, and a checklist at various timepoints during the lifecycle of the innovation platform. Quantitative data obtained at the end of the study related to farmers who were part of the IP. However, since farmers within the IP trained other farmers within their respective blocks on the various SRI practices, we obtained wherever possible by observation or questionnaire similar data provided by the farmers who were part of the IP.

To compare changes in rice productivity and production costs before and after farmers’ participation in the IP, the 24 IP farmer participants completed a questionnaire to quantify their rice yields and income. An observation checklist was also completed during the study to see their level of implementation of various SRI practices. Written consent was obtained for all the participants.

Our primary analysis are descriptive statistics for most questions of interest, alongside inferential statistics to test statistical significance of differences and associations. We used chi-square tests for tests of association between categorical variables. To test for differences in means and proportions, Z-tests were used. A simple cost benefit analysis was done to compare the economic benefits of SRI over conventional farming. All statistical tests were performed at the 5% level of significance. All statistical analysis were performed in STATA (StataCorp, College station, TX, US).

#### Ethics approval statement

This study was approved by the National council for science and technology, Kenya, permit no: NACOSTI/P/18/71198/2644.

## 3. Results

In this section, we begin by describing some important changes in SRI uptake, income levels and then undertake a cost-benefit analysis.

*Table 2* presents a summary of the changes in level of uptake of SRI practice at baseline and end of the study. We observe that there was strong evidence of an increase of each of the practices that comprise SRI (all p-values <0.05). Line planting was the most commonly practiced technique (92%), followed by planting young healthy seedlings (71%) and intermittent watering (62.5%). Less than half of the farmers embraced either mechanization or fertilizer application. The heterogeneity in level of uptake of different practices have brought to the fore discussions about adaptability of the different practices to different farmers [16] or whether farmer perspectives about the technology need to be well understood and perhaps incorporated. Our interactions with farmers often revealed that most farmers completely adopted line planting based on the benefits derived from it, but that not all household members had changed their practices. Most farmers in focus group discussions also suggested that they practiced line planting using the recommended spacing which they found to be more profitable compared to random planting. This may partly suggest that farmers may likely be risk averse at the point of transition from their conventional practices [17].

**Table 2:** Differences in percentage uptake of SRI at baseline and end line.

	Assessment		Test for the difference in proportions	
	Baseline	End line	Z- Test statistic	P-value
Line planting	11.3	91.7	-6.82	< 0.001
Planting young healthy seedlings	47.2	70.8	-3.39	0.001
Intermittent watering	5.8	62.5	-8.45	< 0.001
Manure/Fertilizer application	7.4	45.8	-6.15	< 0.001
Mechanization	4.3	37.5	-5.77	< 0.001

On practices such as manure application, our observations revealed that most farmers do not have access to large quantities of organic manure or biomass to enrich the soil for rice production. While SRI accommodates use of chemical fertilizers, if organic sources of nutrients are insufficient, this is associated with additional costs. Nevertheless, we conjecture that when farmers begin to see the returns from SRI first hand, they can begin to invest more or find alternative solutions.

### Cost benefits analysis of SRI practices in rice production

A cost benefit analysis (CBA) was performed to characterise the economic benefits of SRI to farmers, analysing productivity and revenue returns. Productivity was computed per acre of farmland under rice. We further detailed the cost of operations that were obtained based on the current market rates at the time of data collection. The reference point for the computation of the CBA was 2016 (before the IP formation) and 2019 (following the IP intervention). The result of the CBA is presented in Table 3.

**Table 3:** Estimated cost benefit analysis for rice production under system of rice intensification.

Associated costs (KES)				
Activity	Without SRI		With SRI	
	2016	2019	2016	2019
Land preparation	14,500	19,000	14,500	19,000
Planting	4,900	7,500	4,900	11,110
Fertilizer application	-	-	-	4,750
Weeding	4,000	6,000	4,000	1,300
Pesticides and fungicides	-	-	-	1,650
Irrigation (flooding)	2,000	2,000	2,000	2,300
Bird scaring	1,000	3,000	1,000	3,000
Harvesting and husking	4960	5,420	4,960	8,040
Production costs per acre	31,360	42,920	31,360	51,150
Yield (bags)	24	24	24	41
Revenue and returns on investment. • Paddy rice • Milled rice • Return per shilling invested -paddy • Return per shilling invested -milled	33,600 163,200 0.0714 4.2041	48000 192,000 0.1184 3.4734	33600 163200 0.0714 4.2041	82,000 328,000 0.6031 5.4125

1 USD ≈ KES 100 in 2016/17; * number of bags.

For the conventional farming system (without SRI), there was evidence of a changes in cost of operation which increased by at least 37% (from KES 31,360 to KES 42,920 per acre). One justification for this increase in cost of operation is that the prevailing market prices increased for most inputs of production. On the other hand, we observed a 43% increase in revenue from the sale of paddy (from KES 33,600 to KES 48,000 per acre). This was entirely expected as a result of the increase in market prices per unit of paddy. However, the increase in revenue was only 18% for white milled rice between 2016 and 2019. The narrower margins for milled rice were largely attributed to increased prices of milling. Overall, the return per shilling invested when the farmer sold paddy (KES 0.0714 in 2016 vs KES 0.1184 in 2019) much lower than that realised from the slae of milled rice (KES 4.2041 in 2016 vs KES 3.4734 in 2019). The reduction in return per shilling suggests that there is need to improve efficiency in the value chain for rice farming to remain profitable within the region. Thus there is need to consider implementation of SRI practices.

One of the major concerns in SRI implementation is weed management, where we see that weeding costs are higher without SRI contrary expectation. The main explanation for this is that most farmers had cheaper (family) labour and hand weeders were available by local fabricators. Also, we point out that while mid study data (2017 and 2018) are not presented, the decline was progressive rather than abrupt. We further observe that planting costs under SRI were very high, more than double those in 2016 for SRI, and 53% higher than that undertaken without SRI (*Table 3*). One reason for this is that single seedlings transplantation of seedlings often results in more mortality of seedlings hence there is need for second transplanting which increases the cost of production.

When SRI practices were used for rice production in the irrigation scheme, a massive increase in the cost of operations to the farmer was observed. *Table 3* shows that costs increased by 63% on average from 2016 to 2019 (KES 51,150 per acre in 2019 vs KES 31,360 in 2016). Our observations reveal that the increased cost of production was associated with two factors; i) more input supplies needed when SRI practices were used. For example, farmers cited costs associated with machinery, manure/organic fertilizers, biological control of pests and birds; ii) when fields are not continuously flooded, weed control is a problem necessitating costs associated with hiring labour. Reason (ii) is very important given that in regions such as Madagascar where SRI was first trialled, up to 40% of farmers (mainly the poor) considered SRI to be too labour intensive leading to slightly higher costs [18].

Interestingly, we see in our findings (Table 3) that productivity was higher under SRI than conventional farming (from 24 bags in 2016 to 41 bags (paddy) per acre in 2019). As a result, based of prevailing market rates then we see an increase in revenue of at least 100% for white milled rice. Similarly, the return per shilling invested when SRI practices were used increased from 4.2041 to 4.7393 –a 13% increase.

Based on their own measurements of paddy yields and revenue generated, the actors within the innovation platform were convinced that the participating (IP) farmers were in fact significantly benefitting from increased yield and revenue generation. In the subsequent seasons, new actors have joined the collaboration network and have started producing rice under SRI system within the framework provided by the government extension. Currently the farmer field school (FFS) approach is used for promoting SRI in the neighbouring Kimira irrigation scheme but using the IP experienced farmers as facilitators.

**Fig 2:**
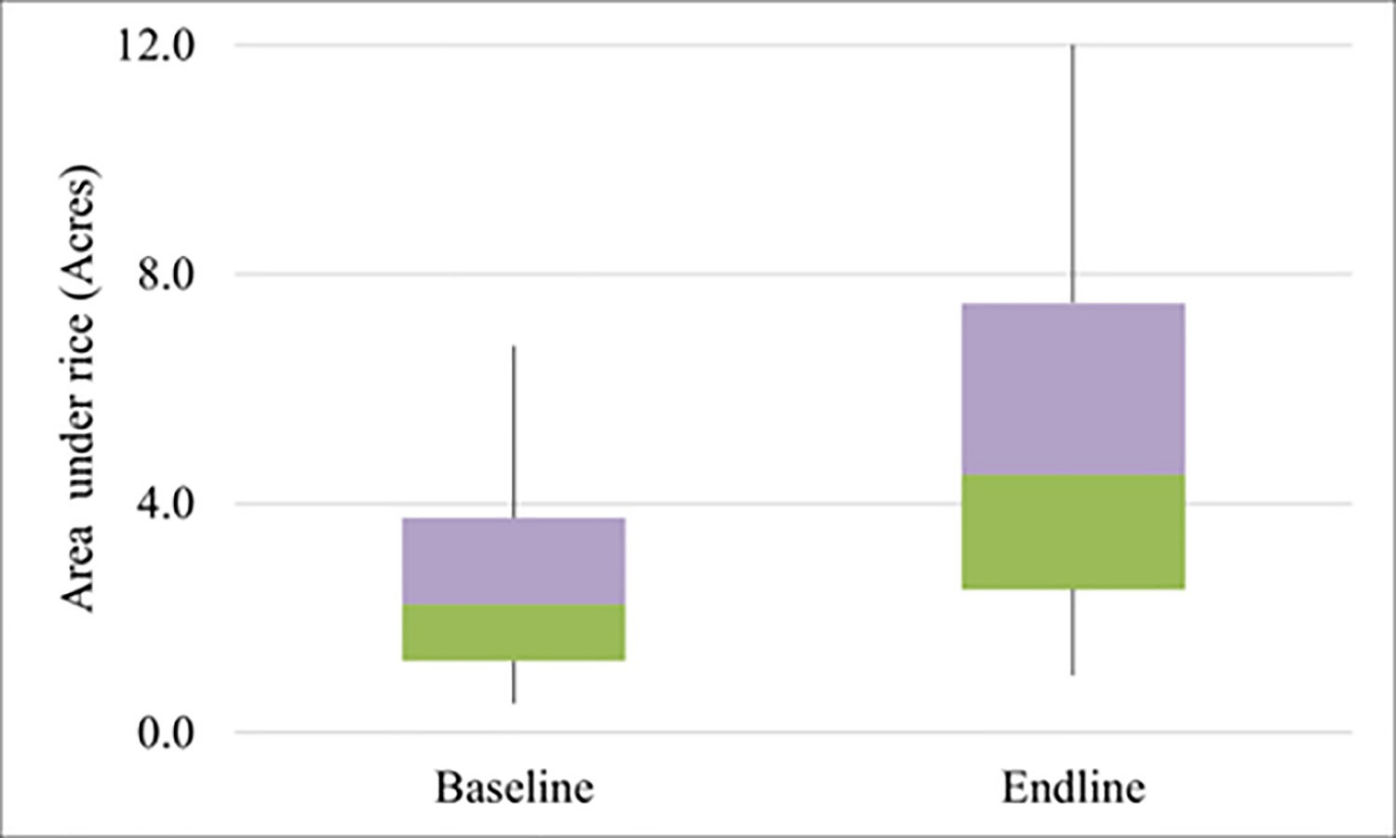
A comparison of farm size between baseline and end line among farmers involved in the innovation platform launched in Oluch irrigation scheme.

We further see in *Fig 2* that over the during when SRI was introduced in Oluch by our IP, the acreage under rice increased significantly; the acreage doubled from baseline (median of 1 acre in 2016 to 2 acres by 2019). Largely, this can be traced to confidence among farmers on the potential benefits of SRI in terms of increasing their yield.

Following the cost-benefit analysis, we also explored among a subset of 40 farmers whether their involvement in an innovation platform was associated with their level of income. *Table 4* shows a summary of results. A Fishers test for association a strong significant association (χ^2^ = 11.814, df = 3, p-value = 0.008) between IP membership and level of income of the farmer. This maybe unsurprising given than IP members had first-hand knowledge from researchers and extensionists when learning from demonstration plots established the IP.

**Table 4:** Cross tabulation of IP participation and level of income.

		Income level of the farmer			Total*
		Low	Average	High	
Membership	IP	5	14	6	25
	Non-IP	10	5	0	15
Total		16	22	2	40

*number of farmers.

## 4. Discussion

In this work we have demonstrated that system of rice intensification indeed caries the promise of increase yield and revenue. Consequently, SRI also carries the promise of improving livelihoods of farmers. Our findings agree with recent research on economic benefits of SRI both in Kenya [2,3] and other countries including India [12,19], Indonesia [17], Ghana [10], Madagascar [20], Tanzania [5].

The CBA results corroborates previous findings of the economic benefits of SRI in Mwea irrigation scheme in Kenya [2,4]. Impressive results were obtained there from the two farmer trials, demonstrating 84 percent and 100 percent increases in paddy harvested from the trial fields [14]. Although we report higher production costs associated with SRI (for instance attributed to labour) like some previous studies [18], we suppose that this is potentially a transient issue or constraint. This is likely to be modified by farmer learning as they find approaches that reduce costs such as local fabrication of weeders by artisans which has actually started off in the area. Consequently, we see that with reduction of costs of production, the technology should become the norm of rice production in the local context. Previous empirical evidence agrees pointing to the fact that with lower costs associated with agricultural innovation, there are less delays on average before the decision to adopt is made [21].

Higher yields and revenues for smallholder farmers are known to be associated with better livelihoods, but also raises an important question whether farmers will spend more time in the crop system. Arguably, the case of smallholder farmers in much of rural Africa is slightly complex, given the multitude of challenges that accost their agricultural productivity endeavours [8]. However, we have demonstrated in another study that rice marketing decisions among these smallholder farmers improved as a result of the innovation platform [13]. This can be a proxy to suggest that the returns are likely to keep more farmers in the cropping system. Nevertheless, it can be argued that findings such as these when replicated in larger studies across different geographical settings, are convincing that given higher returns, farmers are more likely to spend more time in the crop system.

While our study involved a modest number of farmers for training within the innovation platform, we emphasize that this was a reasonable undertaking for the researchers as the most important aspect for IPs to be successful is that they are representative [22,23]. Importantly, this study provides important proof of concept findings that indeed SRI is viable In Oluch, and leads to improved productivity and revenue in other settings in Kenya where rice farming is not done in large scale. In Kenya, all SRI-related studies have been undertaken at Mwea irrigation scheme [4].

The strength of our study lies on the use of the IP to first improve adoption of SRI practices, and secondly the implementation of SRI practices by small-scale farmers to improve yield and subsequently higher returns. The joint action of different stakeholders for the sake of economic or social benefit is generally associated with success of agricultural innovation platforms. The value of the IP lies in the inclusive of stakeholders, from all points in the commodity value chain. It is the multi-stakeholder approach that increases the capacity to solve technical problems and address systemic challenges [24], in our context adoption of SRI practices. Although we do not assess the influence of farmer specific characteristics such as age, physical productivity, education level, information constraints, as important predictors of differences in yield among farmers, we still acknowledge that known individual characteristics that are associated with uptake of SRI [19,25,26] would still explain variations in farmer specific yield and returns on investment. It may be worth exploring the influence of such factors in larger studies. There is also evidence from certain regions of SSA that Socio-economic factors constrain stakeholder engagement and confidence, and that there are limits to the applicability of the innovation platforms [27].

In conclusion, there is need for concerted efforts by the government to upscale system of rice intensification among rice farmers in Kenya, especially leveraging on approaches such as innovation platforms to overcome existing constraints to production and marketing.

## Supporting information

S1 File (DOCX)
