# Correction: A cost benefits analysis of the adoption of system of rice intensification: Evidence from smallholder rice farmers within an innovation platform in Oluch irrigation scheme, Kenya

**DOI:** 10.1371/journal.pone.0314580

**Published:** 2024-11-21

**Authors:** The PLOS ONE Staff

There are errors in the author affiliations. The correct affiliations are as follows:

Matilda A. Ouma^1^, Luke O. Ouma^2^, Justus M. Ombati^3^, Christopher A. Onyango^3^

**1** School of Agricultural and Food Sciences, Jaramogi Oginga Odinga University of Science and Technology, Bondo, Kenya, **2** Biostatistics Research Group, Population Health Sciences institute, Newcastle University, Newcastle upon Tyne, United Kingdom, **3** Department of Agricultural Education and Extension, Egerton University, Egerton, Njoro, Kenya.

The publisher apologizes for the error.
