# Supplementary material for: A cost benefits analysis of the adoption of system of rice intensification: Evidence from smallholder rice farmers within an innovation platform in Oluch irrigation scheme, Kenya
Source: PLoS One. 2024 Jan 2;19(1):e0290759. doi: 10.1371/journal.pone.0290759 (PMC10760909; doi:10.1371/journal.pone.0290759)
Supplement: S1 File — (DOCX) [file pone.0290759.s001.docx]

Supplementary material for “**A cost benefits analysis of the adoption of system of rice intensification: Evidence from a pilot study among smallholder rice farmers in Oluch irrigation scheme, Kenya”**

**by**

Matilda A. Ouma^1*^, Justus M. Ombati^2^, Luke O. Ouma^3^, Christopher A. Onyango^2^

## A Check List for the Cost Benefit Analysis Data

Instruction: Indicate the average quantity of rice produced, consumed, sold and income generated in the last three seasons after intervention.

| **Activity** | **Unit** | **Unit Cost** | **Estimated Cost** | |
| --- | --- | --- | --- | --- |
|  |  |  | **2016** | **2019** |
| Land rent |  |  |  |  |
| Ploughing |  |  |  |  |
| Harrowing |  |  |  |  |
| Rotavating |  |  |  |  |
| **Subtotal** |  |  |  |  |
| Nursery |  |  |  |  |
| Planting (Broadcasting) |  |  |  |  |
| Line Planting |  |  |  |  |
| Planting fertilizer 50kg per acre |  |  |  |  |
| Planting fertilizer application |  |  |  |  |
| Seed (certified) |  |  |  |  |
| Seed (recycled) |  |  |  |  |
| **Subtotal** |  |  |  |  |
| Topdressing fertilizer |  |  |  |  |
| Topdressing fertilizer Application |  |  |  |  |
| Irrigation (Flooding) |  |  |  |  |
| Irrigation (intermittent) |  |  |  |  |
| Manual Weeding |  |  |  |  |
| Mechanical Weeding |  |  |  |  |
| Pesticides & fungicides |  |  |  |  |
| Pesticides & fungicides Application |  |  |  |  |
| Bird Scaring |  |  |  |  |
| Harvesting |  |  |  |  |
| Transport to miller per Bag |  |  |  |  |
| Drying Per Bag |  |  |  |  |
| Milling per kg |  |  |  |  |
| **Subtotal** |  |  |  |  |
| **Production costs per acre** |  |  |  |  |
| Yield paddy Bags per acre (Productivity) |  |  |  |  |
| Number of 2kg tins per bag |  |  |  |  |
| Cost of paddy per 2k tin |  |  |  |  |
| **Revenue paddy per acre** |  |  |  |  |
| Cost of milled 2kg tin |  |  |  |  |
| **Revenue of milled rice per acre** |  |  |  |  |
| Return per shilling invested (paddy) |  |  |  |  |
| Return per shilling invested (milled rice) |  |  |  |  |

## End line Survey Questionnaire

**Section A: Household Characteristics**

1. Farm size? Owned __________, rented__________, Total _________ (acres)
2. Land tenure system: Owned with title deed Owned without title deed Rented
3. What is your income level? Low Average High
4. Do you belong to any social grouping other than the IP and block membership?

Yes No

1. b). If Yes, what benefits do you drive from the group(s)
   1. _______________________________________________________________
   2. _______________________________________________________________
   3. _______________________________________________________________
2. How many stakeholders do you interact with during the period of the rice innovation platform? What was his/her role in the IP? What was the purpose/benefit for the interaction? What was the frequency of the interaction?

|  | Stakeholder’s Name | Interaction  (Y/N) | Who has the stakeholder interacted with in the rice value chain?  (*1 = Farmer 2 = Miller 3 =KOSFIP*  *4 =Extension, 5 =Research, 6 = Trader 7 =Agrovet 8 =Baraka 9= Transporter 10. Admin 11 =Maugo 12=IWUA 13=LBDA 14 =Bayer 15 = KFA 16=Financial*  *17=NIB* 18 = *NARIGP*  *19 =Nyabon 20=Other (specify)* | Frequency  *1 =once*  *2 = twice*  *3=more frequent)* | Purpose of the interaction | How satisfied are you with the interaction?  1 = V. satisfied  2 = Satisfied  3 = Neutral  4 = Unsatisfied  5 = V. unsatisfied |
| --- | --- | --- | --- | --- | --- | --- |
| 1 |  |  |  |  |  |  |
| 2 |  |  |  |  |  |  |
| 3 |  |  |  |  |  |  |
| 4 |  |  |  |  |  |  |

## Observation Checklist for Field Visit

1. **Level of implementation of SRI practices**

Tick appropriately the level at which the farmer implements the following practices (1 represent lowest level of implementation while 5 represent the highest level of implementation)

| **SRI Practices** | | **Implementation of SRI practice** | | | | | |
| --- | --- | --- | --- | --- | --- | --- | --- |
|  |  | **1** | **2** | **3** | **4** | **5** | **Comments** |
| Planting method | Dibbling |  |  |  |  |  |  |
|  | Line planting |  |  |  |  |  |  |
| Planting material | Young healthy seedling |  |  |  |  |  |  |
|  | Overgrown seedlings |  |  |  |  |  |  |
| Irrigation | Flooding |  |  |  |  |  |  |
|  | Intermittent watering |  |  |  |  |  |  |
| Weeding | Mechanical |  |  |  |  |  |  |
|  | Chemical |  |  |  |  |  |  |
|  | Manual |  |  |  |  |  |  |
| Soil fertility | Manure application |  |  |  |  |  |  |
|  | Fertilizer application |  |  |  |  |  |  |
|  | Both manure & fertilizer application |  |  |  |  |  |  |
| Average Yield obtained (Kg/area) | |  |  |  |  |  |  |

## Focus Group Discussion Guide

Some of the questions will emerge to clarify issues which are not clear from the baseline survey.

1. What are the challenges the farmers encounter in implementing rice production practices in Oluch scheme?
2. How do you cope with the mentioned challenges?
3. Who are the stakeholders working with you in the rice production in Oluch Scheme?
4. What are their roles?
5. What is the rice production practices you use in Oluch (probe for system used)?
6. What are the benefits of the practices? (Probe: other farmers learning from++++ them, adoption of the practices by other farmers not in the platform, causal work opportunities for the youth, others?
7. What challenges did you encounter as an actor in working with others?
8. What do you do individually to try and gain more yields?
9. What were the challenges in obtaining the yields?

## Questionnaire for Smallholder Rice Farmers

**Introduction**

The purpose of this baseline survey is to provide a basis upon which change was measured. It will elicit information on the challenges farmers have under conventional rice production practices and farmers’ knowledge gaps in rice intensification practices and also identify relevant stakeholders for establishment of an Innovation Platform to create an opportunity for interactive learning to facilitate uptake of SRI to spur rice productivity in Oluch scheme. Therefore, your honesty in answering the questions was critical to this study. Your responses will be treated with utmost confidence and used strictly for the purpose of this study.

**Section A: Household Characteristics**

1. Age of the farmer: _________________ (years)

2. Sex of household head  Male  Female

3. What is the highest level of formal education attained?

None

Primary

Secondary

Post-secondary

4. Marital status of respondent:

Single

Married

Separated/divorced

Widow/widower

5. Who in your household mainly makes main decisions on how your main household resources are used?

Husband  Wife  Joint (husband, wife and children)

6. Who in your household mainly makes main decisions on agricultural technologies?

Husband  Wife  Joint (husband, wife and children)

b). Who does most of the work on rice on-farm activities?  Men  Women  Both

7. Household size

1-5

6-10

Above 10

8. How long have you been involved in rice farming?

0-5 years

6-10 years

More than 10 years

9. Farm size? Owned __________, rented__________, Total _________ (acres)

10. Land tenure system: Owned with title deed  Owned without title deed  Rented

11. Do you belong to other social groups other than block membership? Yes  No

12. What is the importance/benefits of rice as part of household crop enterprise?

Food security

Income

Fodder (stoves, bran)

Other (specify) ___________________________________________________

13. Indicate the average quantity of rice produced, consumed, sold and income generated in the

last three seasons before intervention.

| Quantity of rice (Kgs) | Produced | Consumed | Sold | Income (Ksh.) |
| --- | --- | --- | --- | --- |
|  |  |  |  |  |
| Season 1 |  |  |  |  |
| Season 2 |  |  |  |  |
| Season 3 |  |  |  |  |

14. What rice production practices/system do you use?

Conventional practices System of rice intensification (SRI)

**SECTION B: Challenges and Benefits of Producing Rice under Conventional Production**

**System**

15. a. What challenges do you experience in producing rice under conventional system?

___________________________________________________________________

___________________________________________________________________

b. What strategies you have adopted to cope with the mentioned challenges?

__________________________________________________________________

__________________________________________________________________

16. What are the benefits of producing rice under conventional system?

________________________________________________________________________

17. Use tick (**√**) to indicate how often you perform the following rice production practices e

on your farm? **Key: not performed = 1, rarely performed = 2, mostly performed = 3**

| Rice production practice | 1 | 2 | 3 |
| --- | --- | --- | --- |
| Planting: Dibbling |  |  |  |
| : Line planting |  |  |  |
| Planting young seedlings |  |  |  |
| Planting old seedlings |  |  |  |
| Irrigation: Flooding |  |  |  |
| : Intermittent watering |  |  |  |
| Weeding: Mechanical |  |  |  |
| : Chemical |  |  |  |
| : Manual |  |  |  |
| Manure application |  |  |  |
| Fertilizer application |  |  |  |

18. How confidently do you carry out the following rice production practices? (***Tick as appropriate)***

| Practice | Least confident | Not Confident | Not sure | confident | Most confident |
| --- | --- | --- | --- | --- | --- |
| 1. Transplanting young seedlings |  |  |  |  |  |
| 2. Planting seedlings per hill |  |  |  |  |  |
| 3. Plant to plant distance- (spacing) |  |  |  |  |  |
| 4. Water management regime |  |  |  |  |  |
| 5. Weeding |  |  |  |  |  |
| 1. Fertilizer application |  |  |  |  |  |
| 7. Manure application |  |  |  |  |  |
| 8.. Harvesting |  |  |  |  |  |
